# Viral OTU Deubiquitinases: A Structural and Functional Comparison

**DOI:** 10.1371/journal.ppat.1003894

**Published:** 2014-03-27

**Authors:** Ben A. Bailey-Elkin, Puck B. van Kasteren, Eric J. Snijder, Marjolein Kikkert, Brian L. Mark

**Affiliations:** 1 Department of Microbiology, University of Manitoba, Winnipeg, Canada; 2 Molecular Virology Laboratory, Department of Medical Microbiology, Leiden University Medical Center, Leiden, the Netherlands; The Fox Chase Cancer Center, United States of America

## Abstract

Recent studies have revealed that proteases encoded by three very diverse RNA virus groups share structural similarity with enzymes of the Ovarian Tumor (OTU) superfamily of deubiquitinases (DUBs). The publication of the latest of these reports in quick succession prevented proper recognition and discussion of the shared features of these viral enzymes. Here we provide a brief structural and functional comparison of these virus-encoded OTU DUBs. Interestingly, although their shared structural features and substrate specificity tentatively place them within the same protease superfamily, they also show interesting differences that trigger speculation as to their origins.

The covalent attachment of ubiquitin (Ub) to protein substrates, i.e., ubiquitination, plays a pivotal regulatory role in numerous cellular processes [1]–[5]. Ubiquitination can be reversed by deubiquitinases (DUBs) [6] and, not surprisingly, various virus groups encode such DUBs to influence ubiquitin-mediated host cell processes [7]–[21]. Some of these viral DUBs resemble proteases belonging to the Ovarian Tumor (OTU) superfamily [22]–[28]. Makarova et al. previously identified OTU proteases as a novel superfamily of cysteine proteases from different organisms [29], and their bioinformatics-based analysis included several of the viral enzymes discussed here. Recently reported structures of these viral DUBs include the OTU domains of the nairoviruses Crimean-Congo hemorrhagic fever virus (CCHFV) [22]–[24] and Dugbe virus (DUGV) [25], the papain-like protease (PLP2) domain of the arterivirus equine arteritis virus (EAV) [26], and the protease (PRO) domain of the tymovirus turnip yellow mosaic virus (TYMV) (Figure 1A–1D) [27], [28]. These viruses are strikingly diverse, considering that nairoviruses are mammalian negative-strand RNA viruses, while the mammalian arteriviruses and plant tymoviruses belong to separate orders of positive-strand RNA viruses.

**Figure 1:**
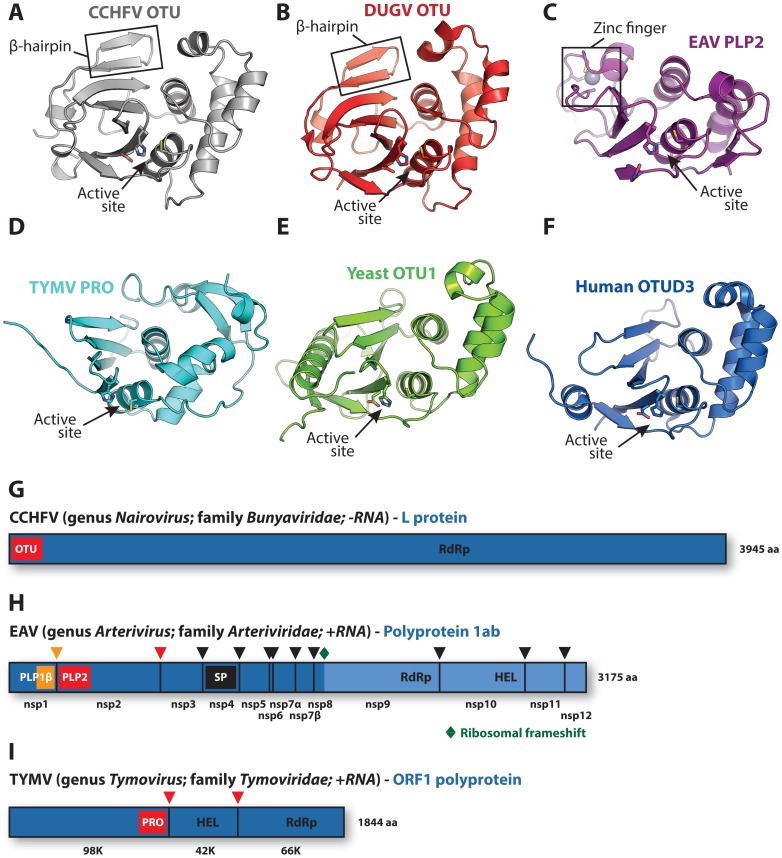
Viral and eukaryotic OTU domain structures and viral protein context. Crystal structures of (**A**) CCHFV OTU (3PT2) [23], (**B**) DUGV OTU (4HXD) [25], (**C**) EAV PLP2 (4IUM) [26], (**D**) TYMV PRO (4A5U) [27], [28], (**E**) yeast OTU1 (3BY4) [57], and (**F**) human OTUD3 (4BOU) [46]. The β-hairpin motifs of CCHFV OTU and DUGV OTU are indicated in boxes in panels **A** and **B**, respectively, and the zinc-finger motif of EAV PLP2 is boxed in panel **C**. Active sites are indicated with arrows. The CCHFV OTU, DUGV OTU, EAV PLP2, and yeast OTU1 domains were crystallized in complex with Ub, which has been removed for clarity. Structure images were generated using PyMol [60]. (**G**) Schematic representation of the CCHFV large (L) protein [61], [62]. A similar organization is found in the DUGV L protein, but is not depicted. The OTU domain resides in the N-terminal region of this protein and is not involved in autoproteolytic cleavage events [48]. (**H**) Schematic representation of the EAV polyprotein 1ab [63]. PLP2 resides in nonstructural protein 2 (nsp2) and is responsible for the cleavage between nsp2 and nsp3 [51]. (**I**) Schematic representation of the TYMV ORF1 polyprotein [50]. PRO resides in the N-terminal product of this polyprotein and is responsible for two internal cleavages [49], [50]. Key replicative enzymes are indicated in **G**, **H**, and **I**. Colored arrowheads denote cleavage sites for the indicated protease domains. HEL, helicase; PLP, papain-like protease; RdRp, RNA-dependent RNA polymerase; SP, serine protease.

Ubiquitination often involves the formation of polyubiquitin chains [1], which can target the ubiquitinated substrate to the proteasome for degradation [2] or modulate its protein–protein interactions, as in the activation of innate immune signaling pathways [3], [4]. Interestingly, several cellular OTU DUBs were found to negatively regulate innate immunity [30]–[33]. Likewise, both nairovirus OTU and arterivirus PLP2 were recently shown to inhibit innate immune responses by targeting ubiquitinated signaling factors [7]–[9], [26], [34], [35]. In contrast to eukaryotic OTU DUBs, both of these viral proteases were found to also deconjugate the Ub-like protein interferon-stimulated gene 15 (ISG15) [7], [36], which inhibits viral replication via a mechanism that is currently poorly understood [37]. Interestingly, coronaviruses (which, together with the arteriviruses, belong to the nidovirus order) also encode papain-like proteases targeting both Ub and ISG15 that were shown to inhibit innate immunity [11]–[13], [38]–[42] but belong to the ubiquitin-specific protease (USP) class of DUBs [6], [43], [44]. The presence of functionally similar, yet structurally different proteases in distantly related virus families highlights the potential benefits to the virus of harboring such enzymes.

The proteasomal degradation pathway is an important cellular route to dispose of viral proteins, as exemplified by the turnover of the TYMV polymerase [45]. Moreover, the degradation of this protein is specifically counteracted by the deubiquitinase activity of TYMV PRO, which thus promotes virus replication [10]. The functional characterization of viral OTU DUBs remains incomplete and future studies will likely reveal additional roles in replication and virus–host interplay.

Polyubiquitin chains can adopt a number of different configurations, depending on the type of covalent linkage present within the polymer [1]. A distal Ub molecule can be linked via its C-terminus to one of seven internal lysine residues present in a proximal Ub molecule via an isopeptide bond. Alternatively, in the case of linear chains, the C-terminus of the distal Ub is covalently linked to the N-terminal methionine residue of the proximal Ub via a peptide bond. While human OTU proteases often show a distinct preference for one or two isopeptide linkage types [46], nairovirus OTUs and TYMV PRO appear to be more promiscuous in their substrate preference [22], [25]. However, like most human OTU proteases, they seem unable to cleave linear polyubiquitin chains in vitro [22], [25], [46]. Arterivirus PLP2 has not been extensively studied in this respect.

It is important to note that many positive-strand RNA viruses, including arteriviruses and tymoviruses, encode polyproteins that are post-translationally cleaved by internal protease domains [47]. Thus, while CCHFV OTU is not involved in viral protein cleavage and its activity seems dispensable for replication (Figure 1G) [48], both arterivirus PLP2 and tymovirus PRO are critically required for viral replication due to their primary role in polyprotein maturation (Figure 1H, 1I) [49]–[53]. Interestingly, while both EAV PLP2 and TYMV PRO can process peptide bonds in cis and in trans [50], [51], PRO does not cleave peptide bonds in linear polyubiquitin chains in vitro [25]. To date, activity of EAV PLP2 towards linear polyubiquitin chains has not been reported.

Based on mutagenesis of putative catalytic residues, arterivirus PLP2 and tymovirus PRO were initially generally classified as papain-like cysteine proteases [51], [54], [55]. Now that crystal structures of these proteases are available, it is possible to search the DALI server [56] in order to identify structurally similar domains. Using the 3-dimensional coordinates of TYMV PRO, the most recently solved structure of a viral OTU protease, such a query identifies structural similarity with eukaryotic OTU DUBs as well as the nairovirus OTU domains and EAV PLP2 (Table 1). A superposition of these viral protease structures with yeast OTU1 [57] further highlights their similarities (Figure 2A–2C), and these comparisons together clearly position them within the OTU DUB superfamily. Sequence comparisons alone were insufficient to demonstrate this conclusively, as the similarity of viral OTU domains to each other and to eukaryotic OTU proteases is very limited and mostly restricted to the areas surrounding the active site residues [29].

**Figure 2:**
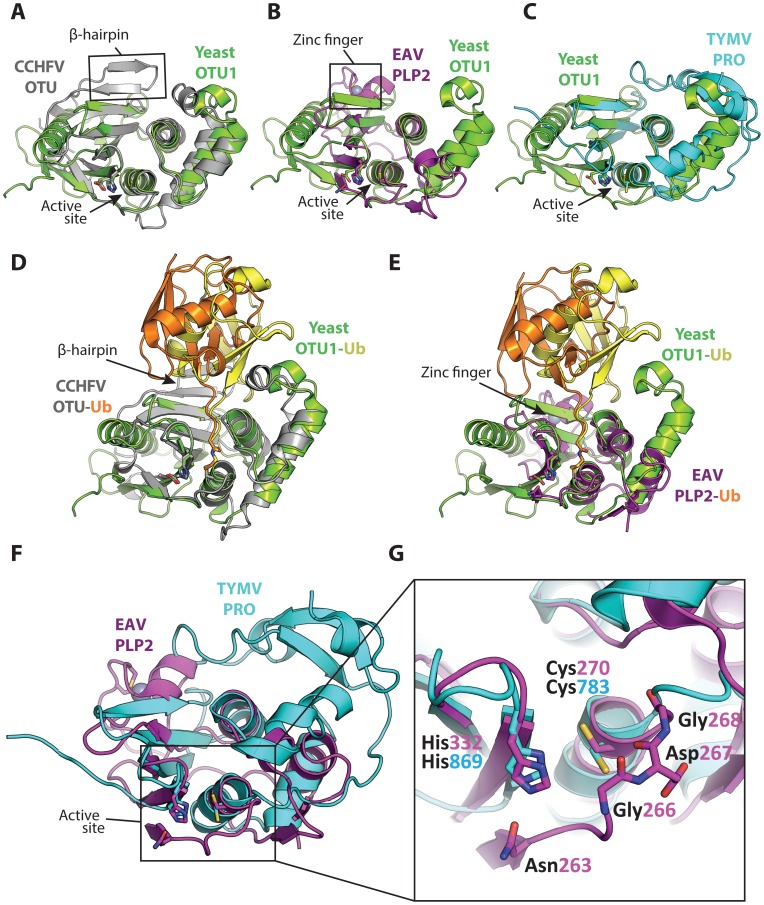
Superpositions of the viral OTU proteases with yeast OTU1 and one another. Superpositions of yeast OTU1 (3BY4) [57] with (**A**) CCHFV OTU (3PT2) [23], RMSD: 1.8 Å over 112 residues, (**B**) EAV PLP2 (4IUM) [26], RMSD: 2.8 Å over 69 residues, and (**C**) TYMV PRO (4A5U) [27], [28], RMSD: 1.4 Å over 76 residues. Superpositions of the yeast OTU1-Ub complex with (**D**) the CCHFV OTU-Ub complex and (**E**) the EAV PLP2-Ub complex, highlighting the difference in the orientation of Ub between the two viral OTU domains versus the eukaryotic yeast OTU1 domain. The Ub that is complexed with yeast OTU1 is depicted in yellow, while the Ub complexed with CCHFV OTU or EAV PLP2 is depicted in orange. (**F**) Superposition of EAV PLP2 and TYMV PRO, RMSD: 2.5 Å over 53 residues. (**G**) Close-up of the active site region (boxed) of the superposition depicted in **F**. Side chains of the catalytic cysteine (Cys270 and Cys783 for EAV PLP2 and TYMV PRO, respectively) and histidine (His332 and His869 for EAV PLP2 and TYMV PRO, respectively) residues are shown as sticks, as well as the active site Asn263 for EAV PLP2. The backbone amide group of Asp267 likely contributes to the formation of the oxyanion hole in the active site of EAV PLP2, yet a functionally equivalent residue is absent in TYMV PRO. The Gly266 and Gly268 residues flanking Asp267 in EAV PLP2 are depicted as sticks as well, for clarity. Note the alternative orientation of the active site cysteine residue of TYMV PRO which, unlike EAV PLP2, was not determined in covalent complex with an Ub suicide substrate. All alignments were generated using the PDBeFOLD server [64], and thus the reported RMSD values differ from those reported in Table 1, in which the DALI server was used. The yeast OTU1, CCHFV OTU, and EAV PLP2 domains were all crystallized in complex with Ub, which has been removed in panels **A**, **B**, **C**, **F**, and **G** for clarity. All images were generated using PyMol [60]. RMSD, root-mean-square deviation.

**Table 1:** Three-dimensional structural alignment of viral OTU domains against selected structures in the Protein Data Bank using the DALI server [56].

DALI Query:	CCHFV OTU	DUGV OTU	TYMV PRO	EAV PLP2
	3PT2 [23]	4HXD [25]	4A5U [27], [28]	4IUM [26]
**Human OTUD3**	14.5; 12%*	14.4; 15%	7.6; 12%	4.2; 13%
4BOU [46]	2.1 Å (123)**	2.1 Å (123)	1.9 Å (85)	2.4 Å (69)
**Yeast OTU1**	11.8; 16%	11.6; 20%	7.3; 12%	5.1; 9%
3BY4 [57]	2.9 Å (126)	2.5 Å (123)	2.3 Å (91)	3.3 Å (81)
**CCHFV OTU**		28.1; 55%	6.8; 15%	4.6; 19%
3PT2 [23]		0.9 Å (157)	3.0 Å (91)	2.6 Å (74)
**DUGV OTU**			6.9; 12%	4.5; 19%
4HXD [25]			2.8 Å (90)	2.6 Å (74)
**TYMV PRO**				3.2; 13%
4A5U [27], [28]				2.8 Å (64)

*z-score (>2 indicates significant structural similarity [59]); % sequence identity.

**Root-mean-square deviation (RMSD) values are indicated, followed by the number of residues used for RMSD calculation in brackets. Value represents the average distance (Å) between alpha carbons used for comparison.

Structural characterization of nairovirus (CCHFV and DUGV) OTU domains and EAV PLP2 in complex with Ub revealed that while these viral proteases adopt a fold that is consistent with eukaryotic OTU DUBs, they possess additional structural motifs in their S1 binding site that rotate the distal Ub relative to the binding orientation observed in eukaryotic OTU enzymes (Figure 2D, 2E) [22]–[26]. In the case of CCHFV OTU, this alternative binding mode was shown to expand its substrate repertoire by allowing the enzyme to also accommodate ISG15. Since TYMV PRO was crystallized in its apo form [27], [28], it remains to be determined whether its S1 site binds Ub in an orientation similar to nairovirus OTU and EAV PLP2 or eukaryotic OTU DUBs.

A remarkable feature of EAV PLP2 is the incorporation within the OTU-fold of a zinc finger that is involved in the interaction with Ub (Figures 1C, 2E). The absence of similar internal zinc-finger motifs in other OTU superfamily members prompted us to propose that PLP2 prototypes a novel subclass of zinc-dependent OTU DUBs [26].

Finally, an interesting structural difference between TYMV PRO and other OTU proteases of known structure is the absence of a loop that generally covers the active site (Figure 2F, 2G). Because of this, TYMV PRO lacks a complete oxyanion hole. It also lacks a third catalytic residue that would otherwise form the catalytic triad that has been observed in other OTU proteases (Figure 2G). Lombardi et al. suggested that the resulting solvent exposure of the active site may contribute to the broad substrate specificity of TYMV PRO [28]. Interestingly, EAV PLP2 also has broad substrate specificity, cleaving Ub, ISG15, and the viral polyprotein, even though it does possess an intact oxyanion hole and an active site that is not solvent exposed. Future work may uncover additional aspects relating to the unusual architecture of the TYMV PRO active site.

The presence of structurally similar proteases, each displaying unique features, in these highly diverse virus groups suggests that their ancestors have independently acquired their respective OTU enzymes. Although their origins remain elusive, one possible scenario is the scavenging of an OTU DUB-encoding gene that directly enabled the ancestral virus to interact with the cellular ubiquitin landscape [29]. The absence of an OTU homologue in other lineages of the bunyavirus family strongly suggests that a nairoviral ancestor acquired an OTU DUB through heterologous recombination. In this scenario, the current differences between the nairoviral and eukaryotic OTU domains would reflect divergent evolution. In the case of arteriviruses, however, it is also conceivable that a preexisting papain-like protease that was initially only involved in polyprotein maturation acquired OTU-like features through a process of convergent evolution. Although rare, such a scenario would account for the limited structural similarity between eukaryotic OTU domains and EAV PLP2, which contrasts with that observed for nairovirus OTU (Figure 2A, 2B; Table 1). For tymoviruses, which encode one (OTU) protease, the existence of related viruses that do not encode a protease domain or that encode one (papain-like) or two (OTU combined with a second papain-like) protease domains complicates the development of a straightforward scenario describing PRO acquisition and evolution [58]. These and other intriguing unsolved questions should be addressed through structural and functional studies of additional OTU-like proteases, be they viral or cellular, the results of which may shed more light on the various scenarios explaining the evolution of viral OTU domains.
